# Study of the Physicochemical and Biological Properties of the Lipid Complex of Marine Microalgae Isolated from the Coastal Areas of the Eastern Water Area of the Baltic Sea

**DOI:** 10.3390/molecules27185871

**Published:** 2022-09-10

**Authors:** Vyacheslav Dolganyuk, Anna Andreeva, Stanislav Sukhikh, Egor Kashirskikh, Alexander Prosekov, Svetlana Ivanova, Philippe Michaud, Olga Babich

**Affiliations:** 1 SEC “Applied Biotechnologies”,, Immanuel Kant Baltic Federal University, A. Nevskogo Street 14, 236016 Kaliningrad, Russia; 2 Department of Bionanotechnology, Kemerovo State University, Krasnaya Street 6, 650043 Kemerovo, Russia; 3 Laboratory of Biocatalysis, Kemerovo State University, Krasnaya Street 6, 650043 Kemerovo, Russia; 4 Natural Nutraceutical Biotesting Laboratory, Kemerovo State University, Krasnaya Street 6, 650043 Kemerovo, Russia; 5 Department of General Mathematics and Informatics, Kemerovo State University, Krasnaya Street 6, 650043 Kemerovo, Russia; 6 Institut Pascal, Université Clermont Auvergne, CNRS, Clermont Auvergne INP, F-63000 Clermont-Ferrand, France

**Keywords:** *Chlorella vulgaris*, *Arthrospira platensis*, minimum inhibitory concentration, lipid–pigment complex, antimicrobial activity

## Abstract

The Baltic Sea algae species composition includes marine euryhaline, freshwater euryhaline, and true brackish water forms. This study aimed to isolate a lipid–pigment complex from microalgae of the Baltic Sea (Kaliningrad region) and investigate its antimicrobial activity against Gram-positive and Gram-negative bacteria. Microalgae were sampled using a box-shaped bottom sampler. Sequencing was used for identification. Spectroscopy and chromatography with mass spectroscopy were used to study the properties of microalgae. Antibiotic activity was determined by the disc diffusion test. Lipids were extracted using the Folch method. Analysis of the results demonstrated the presence of antimicrobial activity of the lipid–pigment complex of microalgae against *E. coli* (the zone diameter was 17.0 ± 0.47 mm and 17.0 ± 0.21 mm in *Chlorella vulgaris* and *Arthrospira platensis*, respectively) and *Bacillus pumilus* (maximum inhibition diameter 16.0 ± 0.27 mm in *C. vulgaris* and 16.0 ± 0.22 mm in *A. platensis*). The cytotoxic and antioxidant activities of the lipid complexes of microalgae *C. vulgaris* and *A. platensis* were established and their physicochemical properties and fatty acid composition were studied. The results demonstrated that the lipid–pigment complex under experimental conditions was the most effective against *P. pentosaceus* among Gram-positive bacteria. Antimicrobial activity is directly related to the concentration of the lipid–pigment complex. The presence of antibacterial activity in microalgae lipid–pigment complexes opens the door to the development of alternative natural preparations for the prevention of microbial contamination of feed. Because of their biological activity, Baltic Sea microalgae can be used as an alternative to banned antibiotics in a variety of fields, including agriculture, medicine, cosmetology, and food preservation.

## 1. Introduction

Many studies have reported on the antimicrobial activity of lipid–pigment complexes (LPCs) over several years [1,2,3,4,5]. This important feature provides LPCs with the potential to be applied in various fields, such as agriculture, medicine, cosmetics, food preservation, and in particular, as an alternative to banned antibiotics [6]. It was reported that the antimicrobial activity of LPCs depends on both the chain length and the degree of fatty acid unsaturation [7,8]. LPCs belong to the group of secondary metabolites, which are used to test biological activity after isolation and purification from minor bases [9]. The production of bioactive compounds from microalgae can be beneficial for the pharmaceutical industry [10,11].

Fatty acids have diverse and potent biological properties; for example, free fatty acids (FFAs) can kill or inhibit bacterial growth. Many organisms use the antibacterial properties of free fatty acids as a defense against parasitic or pathogenic bacteria. Although the FFA antibacterial mechanism is still poorly understood, their main target is the cell membrane, where FFAs disrupt the electron transport chain and oxidative phosphorylation [3].

As well as interfering with the cell’s energy production, the FFA’s action can also result in enzyme activity inhibition, impaired absorption of nutrients, formation of peroxidation and autooxidation products, or direct lysis of bacterial cells [4]. Their broad-spectrum, nonspecific mechanism of action and their safety make them attractive antibacterial agents for various applications in medicine, agriculture, and food preservation, especially where the use of conventional antibiotics is undesirable or prohibited. Moreover, the development of inducible phenotypes resistant to FFA has fewer negative consequences than traditional antibiotics. The possibility of commercial or biomedical use of antibacterial LPCs, especially those derived from microalgae, is being discussed.

**Figure 1:**
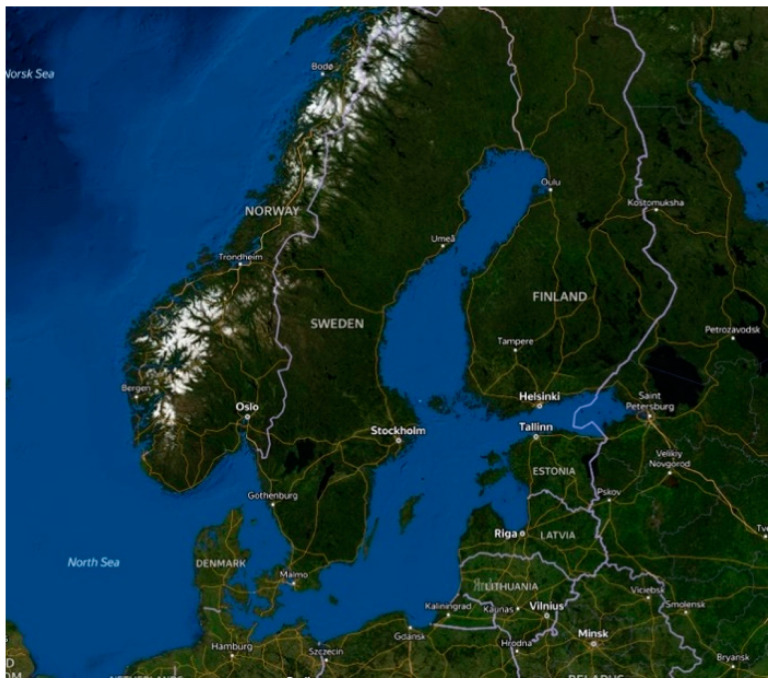
Zoning of the Baltic Sea (Source: https://yandex.ru/maps/ accessed on 11 May 2022).

The relatively low salinity of the Baltic Sea limits the development of both marine and freshwater species; therefore, the quantitative and qualitative composition of green algae is depleted. The species composition of sea algae includes marine euryhaline, freshwater euryhaline, and true brackish water forms. Within the Gdansk Basin (Figure 1), 458 species, varieties, and forms of phytoplankton from seven taxonomic divisions are noted. The greatest species diversity is typical for green algae and diatoms [12]. The greatest phytoplankton species diversity is typical for coastal areas, while in deep-water areas, phytoplankton is not so widely represented [13,14].

Microalgae generate biologically active forms of substances that greatly influence the microbiota of the Earth. Their influence on the development and growth of pathogenic microorganisms makes them a modern form of natural antibacterial agent [15,16]. *C. vulgaris* and *A. platensis* are active producers of proteins, carbohydrates, lipids, and vitamins. The ratio of these compounds is easily adjusted with the change in cultivation conditions: dry biomass grown on ordinary mineral media contains 40–55% protein, 35% carbohydrates, 5–10% lipids, and up to 10% mineral substances, while the biomass obtained on the medium with a different concentration of components can have the following composition: 9–88% protein, 5–86% lipids, 6–38% carbohydrates. Phycocyanin, a part of the *A. platensis* protein fraction, is the most potent natural immunostimulant.

This study aimed to isolate an LPC from microalgae of the Baltic Sea (Kaliningrad region) and investigate its antimicrobial activity against Gram-positive (*Bacillus pumilus*, *Leuconostoc mesenteroides* subsp. Mesenteroides, *Pediococcus pentosaceus*) and Gram-negative (*Escherichia coli*) bacteria.

## 2. Results

### 2.1. Isolate Identification

**Figure 2:**
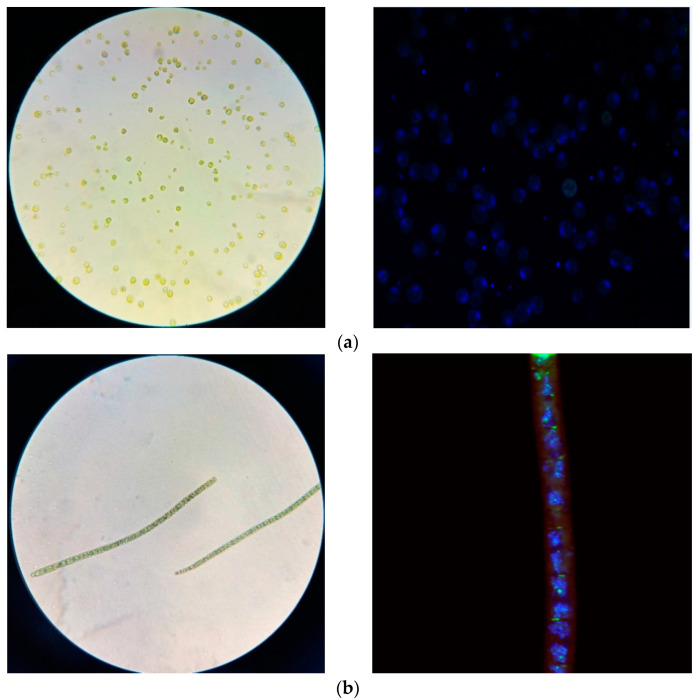
Light microscopy and fluorescence micrography (Bodipy and Dapi staining) of lipid inclusions in the cells of (**a**) *C. Vulgaris*; (**b**) *A. platensis*.

To identify isolates from the enrichment culture strains of microorganisms (microalgae), partial sequences of the 18S and/or 16S rRNA gene were determined, after which a comparative analysis was performed with the known sequences from the Genbank database. The results of a comparative analysis of the 18S rRNA gene sequence indicated that the following microalgae were isolated from natural sources (soil, water, sand): *Chlorella vulgaris* and *Arthrospira platensis* (Figure 2).

### 2.2. Physicochemical Properties of Microalgae

The results of studying the physicochemical properties of microalgae are presented in Table 1.

The lowest optical density (OD750 = 0.59 ± 0.01) after 7 days of cultivation was observed in samples of microalgae *C. vulgaris*, and the highest value (OD750 = 0.86 ± 0.02) was in samples of microalgae *A. platensis*. The density of the *A. platensis* microalgae suspension samples after 7 days of cultivation was 928.48 ± 27.75 kg/m^3^, which is significantly higher than the density of the *C. vulgaris* microalgae suspension samples (909.34 ± 27.42 kg/m^3^).

When studying the dynamic viscosity of suspensions, it was found that the *C. vulgaris* microalgae suspension samples after 7 days of cultivation reached a higher viscosity than the *A. platensis* microalgae suspension samples. The dynamic viscosity of the *C. vulgaris* suspension was (1.13 ± 0.03)·10^−3^ Pa·s, and that of the *A. platensis* suspension was (0.96 ± 0.02)·10^−3^ Pa·s. A higher content of dry matter among the studied samples was noted for the *A. platensis* microalgae biomass (0.59 ± 0.01%); the content of dry matter in the *C. vulgaris* microalgae biomass was no more than 0.49 ± 0.01%.

Suspension samples of the studied microalgae differed in the value of active acidity (from 8.4 to 8.7 and from 9.6 to 11.6 for *C. vulgaris* and *A. platensis*, respectively), but all microalgae grew and actively developed in an alkaline medium. The qualitative and quantitative content of the lipid fraction of the samples of the lipid complex of microalgae are presented in Table 2.

The lipid complex of microalgae *C. vulgaris* samples contained more chlorophyll and other impurities (16.3 ± 0.1 and 57.9 ± 0.8, respectively). Scharff et al. [17] showed that the lipid complex of microalgae *C. vulgaris* contains only triglycerides (49 ± 0.7), and in small amounts (16.9 ± 0.6), fatty acids [17]. Considering that in our studies, not only were triglycerides and fatty acids obtained, but also neutral lipids, polar lipids, unsaponifiable substances, and chlorophyll, we can conclude that our results indicate a more efficient way of isolating and purifying the lipid complex in the course of the studies.

The results of a detailed analysis of the fatty acid composition of the lipid fraction of samples of the lipid complex of microalgae are presented in Table 3.

**Figure A1:**
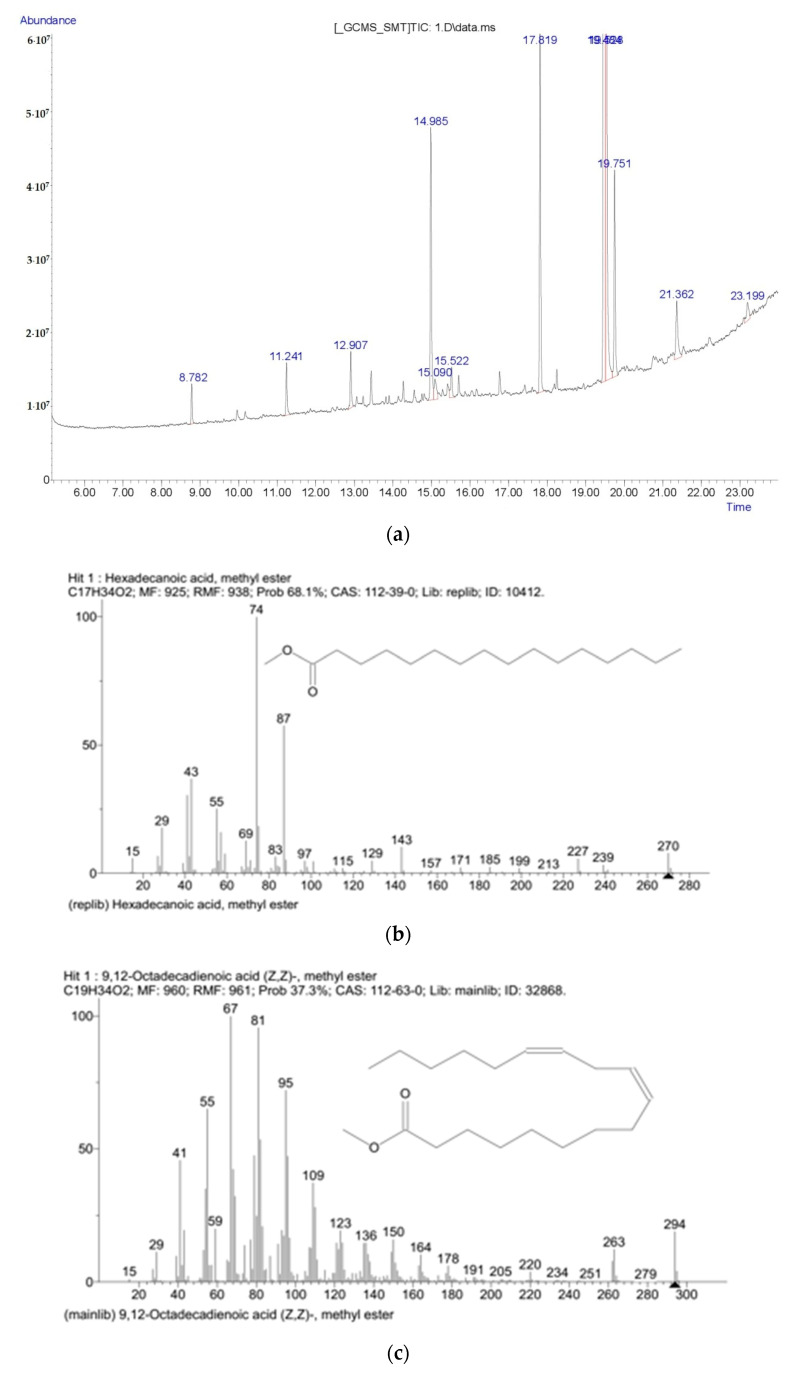

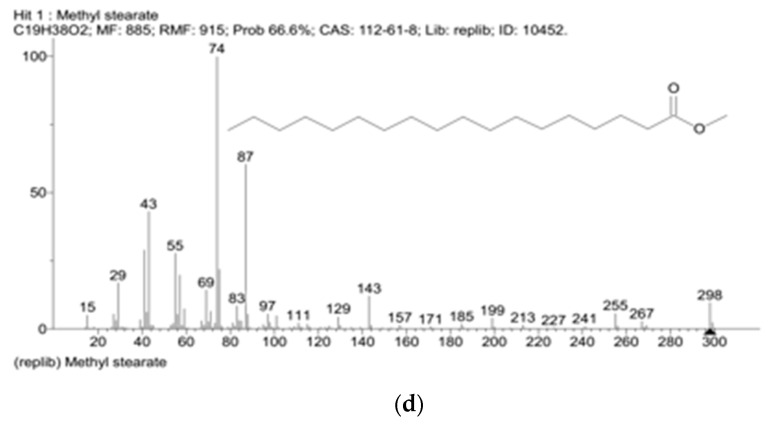
Typical chromatogram (**a**) for FAMES and qualitative GC-MS analysis; (**b**) hexadecenoic acid; (**c**) 9,12-Octadecadienoic acid; (**d**) methyl stearate.

Typical chromatogram and MS spectrum for FAMEs present on Figure A1.

### 2.3. Antimicrobial Activity

This study preliminarily evaluated the bactericidal and bacteriostatic activity of the lipid complex in vitro. We will be able to select pharmacological models that correspond to the studied biological targets in vitro in future in vivo tests [18]. Table 4 and Table 5 show the size of the inhibition zone (mm) of the test strains in vitro by the lipid complex.

*C. vulgaris* and *A. platensis* purified LPC was effective against *E. coli* at the highest concentration (10.0 μg/disc), and the inhibition zone for *C. vulgaris* and *A. platensis* was 17.0 ± 0.47 mm and 17.0 ± 0.21 mm, respectively. However, inhibition was still observed at a 5.0 μg/disc concentration but was lower, on average, by 6.1 ± 0.13 mm for *C. vulgaris* and 7.0 ± 0.23 mm for *A. platensis*. It is established that the greatest increase in antimicrobial activity was observed for *C. vulgaris* and *A. platensis* lipid complexes in the concentration range from 5.0 to 7.5 μg/disc. This is due to the fact that this concentration range is the most rational for the manifestation of antimicrobial activity in the studied lipid complexes. With a concentration increase to 10.0 μg/disc, antimicrobial activity increased, but with a smaller step.

The zone of inhibition of *B. pumilus* under the influence of the *C. vulgaris* lipid complex is shown in Table 4 and Table 5. LPC showed antimicrobial activity against *B. pumilus* at 7.5 and 10.0 μg/disc with a maximum inhibition area of 16.0 ± 0.27 mm in the case of *C. vulgaris* and 16.0 ± 0.22 mm in the case of *A. platensis*. The minimum concentration of 5.0 μg/disc had an average value of the inhibition zone of 6.2 ± 0.23 mm (*C. vulgaris*) and 8.0 ± 0.14 mm (*A. platensis*). The growth inhibition reaction of *L. mesenteroides* induced by the purified lipid fraction of microalgae is shown in Table 1 and Table 2. Purified LPC actively inhibited *L. mesenteroides* at a dose of 7.5 μg/disc and 10.0 μg/disc, with a maximum inhibition area, on average, of 14.5 ± 0.15 mm and 17.0 ± 0.65 mm (*C. vulgaris*) and 15.6 ± 0.23 mm and 18.0 ± 0.24 (*A. platensis*). The 5.0 μg/disc concentration showed a low result, with the area of the zone of inhibition, on average, being 7.6 ± 0.14 mm (*C. vulgaris*) and 7.6 ± 0.14 mm (*A. platensis*). The results obtained had a high level of reliability; the probability of antimicrobial activity of the tested organic extract was *p* < 0.05, which was confirmed by the degree of error (error), using statistical modeling at n = 3.

**Figure 3:**
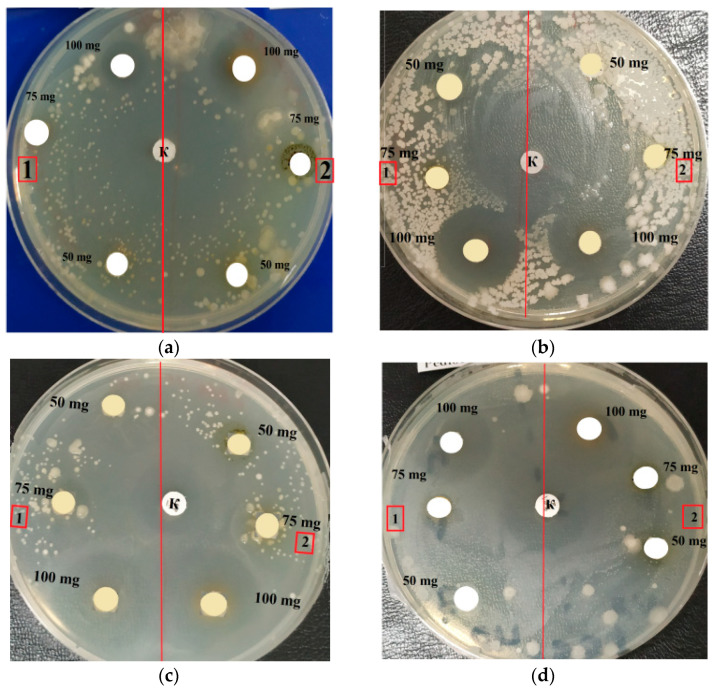
Inhibition zones (**a**) *E. coli*; (**b**) *L. mesenteroides*; (**c**) *B. pumilus*; (**d**) *P. pentosaceus: 1—A. platensis; 2—C. vulgaris*.

The literature data analysis showed that the antimicrobial activity of LPC is the result of the influence of palmitic, α-linolenic, and oleic acids, because the activity of fatty acids increases with an increase in the number of double bonds in the acid molecule [19,20]. The pronounced inhibition of four different types of bacteria is illustrated in Figure 3. *A. platensis* and *C. vulgaris* lipid fraction samples were tested at three concentrations of 5.0, 7.5, and 10.0 μg/disc. Discs with ampicillin (10 μg/disc) were used as controls to assess the inhibition zones. It was found that almost all metabolites of microorganisms in high concentrations exhibited microbial inhibition. Microalgae *A. platensis* and *C. vulgaris* (Figure 3) suppressed the growth of pathogenic and opportunistic microorganisms. The largest zone of inhibition was observed for *E. coli* and *P. pentosaceus*. The activity of *A. platensis* and *C. vulgaris* against these microorganisms was approximately at the same level.

### 2.4. Minimum Inhibitory Concentration (MIC)

**Figure 4:**
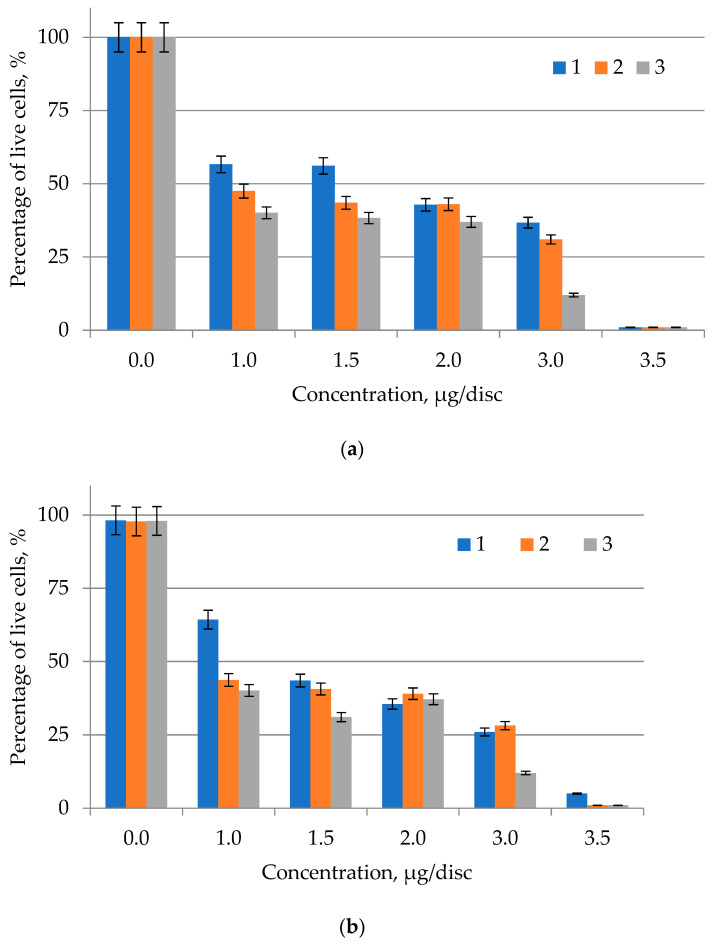
Dependence of the minimum inhibitory concentration (MIC) of the purified lipid–pigment complex and the inhibition level (IL) (**a**)—*C. vulgaris;* (**b**)—*A. platensis;* 1—*E. coli*; 2—*B. pumilus;* 3—*L. mesenteroides*.

The MIC evaluation criteria consisted of a range of LPC concentrations and the same test cultures that were tested for antimicrobial activity (Figure 4). The obtained results were presented in a digital array according to the above diagram; the wavelength of the microplate reader was 600 nm. According to the obtained data, the LPC concentration had lower values when the growth of bacterial cultures was inhibited, i.e., the level of inhibition was higher under microplate conditions. The diagram illustrates the bacterial mass growth inhibition in more detail. The level of inhibition was caused by the reaction of LPC from microalgae against test bacteria (Figure 4). The concentration of 3.0 μg/disc of lipid-containing substance shows a high level of bacterial growth inhibition. The minimum concentration (1.0 μg/disc) showed a low level of inhibition.

The data show the reaction of Gram-positive bacteria *B. pumilus* and *L. mesenteroides* with different concentrations of purified LPC from *C. vulgaris* and *A. platensis* analyzed using a plate reader (Figure 4). The experimental concentrations of LPC were 1.0, 1.5, 2.0, 3.0 μg/disc; solvent methanol was used as a positive control, while a selected bacterial culture in LB culture medium was used as a negative control. The level of inhibition of bacterial growth increased with increasing concentration. The graphical data represent an expressive picture obtained under the conditions of the experiment. Figure 4 shows the reading pattern of the inhibition level caused by the LPC reaction in suppressing Gram-positive bacteria. No apparent differences suggest that the inhibitory activity of lipid-containing compounds was more pronounced against Gram-positive or Gram-negative bacteria.

Therefore, it can be seen from the diagram that the minimum concentration that can inhibit the growth of bacteria is 3.0 μg/disc (the optical density level was 0.260 a.u.). It was proven that purified LPC of *C. vulgaris* and *A. platensis* at a concentration of 3.0 μg/disc was an effective antibacterial agent against Gram-positive bacteria *B. pumilus* and *L. mesenteroides*. The MIC of microalgae lipids against *E. coli* was 2.0 μg/disc, because neutral lipids in the lipid fraction of microalgae, such as triacylglycerides, proved to be the most effective compound as antimicrobial agents. The MIC of algae against *S. aureus* was 0.5 μg/disc, and the maximum MIC was 1.25 μg/disc [21]. The MIC against *S. aureus* observed in this experiment was almost the same as in the previous study by Little et al. [22], which was in the range of 1.6–5.0 μg/disc.

### 2.5. Determination of the Cytotoxicity of Lipid Complexes of Microalgae

**Figure 5:**
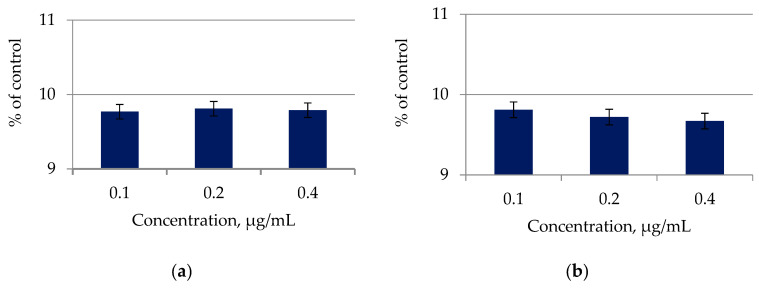
Effect of lipid complexes of microalgae (**a**) *C. vulgaris* and (**b**) *A. platensis* on the skin melanoma cell line SK-MEL-2 after 48 h of incubation.

A trypan blue test was performed to determine changes in the viability of the skin melanoma cell line SK-MEL-2 after contact with lipid complexes of microalgae *C. vulgaris* and *A. platensis* after 48 h of exposure. Figure 5 presents the results of determining the cytotoxicity of lipid complexes of microalgae.

The isolated and purified lipid complexes of microalgae *C. vulgaris* and *A. platensis* samples had a significant toxic effect on the skin melanoma cell line SK-MEL-2 in the concentration range from 0.1 to 0.4 μg/mL, reaching values up to 9.7% of control (Figure 5), which indicated their high cytotoxicity. The proapoptotic activity of samples of lipid complexes of microalgae *C. vulgaris* and *A. platensis* was investigated in relation to nuclear condensation of skin melanoma cells SK-MEL-2 using fluorescence microscopy. Fluorescence microscopy showed nuclear changes such as chromatin condensation, nuclear fragmentation, and apoptotic body formation in the SK-MEL-2 skin melanoma cell line. It was found that melanoma cells increase the early stages of apoptosis by 35–45% and the stage of late apoptosis by 38–20%, respectively. According to the research results, it can be concluded that lipid complexes of microalgae *C. vulgaris* and *A. platensis* can cause apoptosis of the skin melanoma line SK-MEL-2 in vitro.

### 2.6. Determination of Antioxidant Properties of Lipid Complexes of Microalgae

Table 6 presents the results of the analysis of the antioxidant activity of lipid complexes obtained from the microscopic algae biomass.

Lipid complexes of the studied microalgae had significant antioxidant activity (Table 6), which is practically the same for both studied samples.

## 3. Discussion

In the process of studying the physicochemical properties of microalgae, it was found that both objects of study (*C. vulgaris*, *A. platensis*) had no odor at the initial stages of cultivation, and with an increase in the duration of the biomass production process, a faint herbaceous odor appeared. The color of the suspension during cultivation was green in all studied samples. With an increase in the cultivation process duration, the optical density, density, and dynamic viscosity increased, which indicates that the accumulation of biomass occurred during the cultivation of microorganisms.

The effective concentration of lipid–pigment complex (LPC) isolated from *C. vulgaris* was 3.0 μg/disc at an optical density of 0.367 a.u., which showed the best inhibitory activity against Gram-negative bacteria *E. coli*. High inhibitory activity was expressed in suppressing the growth of bacterial colonies and the minimum value of optical density. The growth inhibition of pathogenic and opportunistic microorganisms was not observed at a concentration of 0.5 μg/disc. Therefore, it should be noted that the concentration of 3.0 μg/disc showed the highly effective antibacterial activity of the lipid–pigment complex of *C. vulgaris* against Gram-negative bacteria *E. coli*. Protein concentrate, lipid–pigment, and carbohydrate–mineral complexes were isolated and studied for the first time from samples of microalgae growing in the Baltic Sea in Kaliningrad region territory. The MICs of lipid–pigment complex of microalgae *C. vulgaris* and *A. platensis* obtained in the experiment are highly novel and have not been previously studied. It was established for the first time that the protein concentrate, lipid–pigment, and carbohydrate–mineral complexes from microalgae exhibited antimicrobial activity. The physicochemical properties of these microalgae were studied for the first time.

The lipid complex of microalgae contained neutral lipids, triacylglycerides, fatty acids, polar lipids, unsaponifiables, chlorophyllides, and other impurities (Table 2). The largest number of fatty acids, polar lipids, and unsaponifiables was found in the lipid complex of *A. platensis* microalgae samples. The lipid fraction of the lipid complex of microalgae *C. vulgaris* and *A. platensis* included essential fatty acids (Table 3), valuable for the production of feed additives for animal husbandry: myristic, palmitic, oleic, stearic, and linoleic acids. The content of fatty acids in the samples of the studied algae was comparable, with the exception of palmitic and oleic acids. Table 4 and Table 5 show zones of inhibition of purified LPC obtained from *C. vulgaris* and *A. platensis* at various concentrations, antimicrobial activity against a Gram-negative *E. coli* strain was demonstrated.

Similar results of the *C. vulgaris* antimicrobial activity study are presented in [23,24,25,26]. It was found that the lipid extract of *C. vulgaris* at a concentration of 15.0 µg/disc exhibited antimicrobial activity against the tested organisms. The diameters of the zones of inhibition were as follows: *A. niger* 51 mm; *C. albicans* 47 mm; *E. coli* 24 mm; *S. aureus* 25 mm. Lipid extract of *C. vulgaris* showed antibacterial activity against Gram-negative (*E. coli*) and Gram-positive (*S. aureus*) bacteria. In other studies, extracts of green unicellular algae demonstrated pronounced antagonistic activity against numerous opportunistic and pathogenic bacteria [27]. The *A. platensis* lipid fraction also showed an inhibitory effect against pathogenic and opportunistic microorganisms (including *E. coli*) [17]. These results confirm the potential of *C. vulgaris* lipid extracts for the production of natural fungicides and bactericides. However, the study [28] investigated only the extract of *Chlorella vulgaris* grown on a postfermentation filter of a biogas plant with vinasse and corn silage, while we isolated microalgae *C. vulgaris* and *A. platensis* from the Baltic Sea in the Kaliningrad region. Only the lipid–pigment complex was used as an extract.

Previously, similar properties of *C. vulgaris* were studied during cocultivation with *A. brasilense*, which further enhanced the antimicrobial activity of *C. vulgaris* [18,19,29]. The literature data analysis showed that the antimicrobial activity of LPC is the result of the influence of palmitic, α-linolenic, and oleic acids [18,19,20]. Some authors indicate that extraction with methanol yields a higher product yield than hexane and ethyl acetate [18,19,20,29,30]. Chloroform and methanol were the most effective organic solvents for lipid extraction, preserving antibacterial activity [21,31,32].

German-Báez et al. [33] discovered that residual microalgae biomass, a byproduct of biofuel production, can be used to develop sustainable nutraceuticals and functional foods. Galactopyranoside esters exhibited weak-to-moderate activity against Gram-positive *S. aureus* in [34]. Esters of galactopyranosides showed promising antifungal activity [35,35]. Muhammad et al. [36] demonstrated that rhamnopyranoside esters, which are both hydrophilic and lipophilic, have a broader range of applications, including anticancer activity. According to the findings of all studies, the moderate antimicrobial efficacy of isopropylidene-protected rhamnopyranosides may be due to their conformational distortion, lower mildness, and smaller dipole moments [36].

When studying the effect of the lipid complex of microalgae on the proliferative activity of cancer cells (the ability to form a colony from one cell) a significant inhibition of proliferation in the line of treated cancer cells was noted. The ability to form colonies of tumor cells treated with samples of lipid complexes of microalgae was reduced by at least 50%, while for healthy cell lines it was reduced by only 20%. According to these results, samples of lipid complexes of microalgae *C. vulgaris* and *A. platensis* had the potential to inhibit the formation of double tumor colonies in vitro compared to normal cells. SK-MEL-2 skin melanoma cell staining with acridine orange/ethidium bromide was evaluated to detect nuclear changes and the formation of apoptotic bodies to prove the presence of cytotoxic activity and the inhibition of cancer cells, and the proliferation of microalgal lipid complexes by samples.

## 4. Materials and Methods

### 4.1. Materials

The materials were LB agar medium for the cultivation of microorganisms, and analytically pure reagents: NaCl (JSC LenReactiv, Saint Petersburg, Russia), chloroform (JSC LenReactiv, Saint Petersburg, Russia), methanol formazin (JSC LenReactiv, Saint Petersburg, Russia), hydrazine sulfate (JSC LenReactiv, Saint Petersburg, Russia), urotropin (JSC LenReactiv, Saint Petersburg, Russia), and zinc selenide (JSC LenReactiv, Saint Petersburg, Russia).

### 4.2. Microalgae Samples

Samples of microalgae were collected from the Baltic Sea (Kaliningrad, Russia) in June 2020. A box-shaped bottom sampler developed at the Institute for Biology of Inland Waters of the Russian Academy of Sciences (IBIW) (Borok, Russia), covering a square area of the bottom 160 × 160 mm in size with a maximum immersion depth of 440 mm in bottom sediments, was used for this purpose; a 400 mm-length sample was taken. Immediately after transportation to the shore, test cores were taken using plastic tubes with an inner diameter of 45 mm. The tubes were sealed at both ends and stored in an upright position at +4 °C. In the laboratory, the core was cut lengthwise and halved using two thin stainless-steel plates inserted into the cut. The halves of the core were then divided into transverse samples (slices) with a step of 5–10 mm. The concentration of bacterial cells was 107–108 CFU/mL. All samples were stored at −20 °C in the dark, in plastic bags with squeezed air, from which microalgae samples were taken for research.

Further, pure microalgae cultures were isolated, and microalgae strains that can actively accumulate biomass and target products (lipids, proteins, and carbohydrate–mineral complex) and are suitable for cultivation in laboratory conditions were identified. The harvested microalgae were washed to remove impurities and cultured in 500 mL Erlenmeyer laboratory flasks. The cultivation was carried out on an orbital shaker Heidolph Unimax 1010 a(Heidolph Instruments Gmbh & Co Kg, Schwabach, Germany)t 118 rpm and T = 22 °C. The algae were dried in a drying oven at T = 40 °C (Memmert, Büchenbach, Germany). Before extraction, the samples were stored at T = 4 °C.

To identify isolates from the enrichment culture strains of microorganisms (microalgae), partial sequences of the 18S rRNA gene were determined. DNA from the samples was isolated using DNeasy Plant Pro Kit (Quagen, Limburg, Germany). The primer annealing regions corresponded to forward primer 5′-AACCTGGTTGATCCTGCCAG-3′ and reverse primer 5′-CACCAGACTTGCCCTCCA-3′. The samples were amplified by the qPCRmix-HSreaction mixture (Eurogen, Moscow, Russia) using C1000 Touch system (BioRad, Hercules, CA, USA) and were cloned into pAL2T-vector (Eurogen, Moscow, Russia). After that, the recombinant vectors were sequenced by M13 primer system (Eurogen, Moscow, Russia) using 3500 Genetic Analyzer (Applied Biosystems, Waltham, MA, USA). The sequence processing was performed with software CLC Genomics Workbench (Quagen, Germany). The comparative analysis was performed with the known sequences from the Genbank database. The results of a comparative analysis of the 18S rRNA gene sequence indicated that the following microalgae were isolated from natural sources (soil, water, sand): *Chlorella vulgaris* and *Arthrospira platensis*.

### 4.3. Investigation of the Physicochemical Properties of Microalgae

Active acidity, optical density of the suspension, density, dynamic viscosity, dry matter content, color, and odor of the suspension were chosen as the main indicators characterizing the physicochemical properties of the samples under study. The optical density of the suspension at different stages of cultivation was studied using a UV-3600 two-beam spectrophotometer (Shimadzu, Kyoto, Japan). The measurements were carried out at a wavelength of 750 nm in 1.0 cm glass cuvettes. Distilled water was used as a control. If the instrument readings exceeded the operating range, the samples were diluted with distilled water. The dry weight of the samples was determined by filtration through paper filters previously dried in a drying oven to constant weight. Suspension was sampled with sterile medical syringes with a volume of 100 mL, then was filtered under vacuum using a Bunsen flask and a Büchner funnel. After filtration, the filters were washed with distilled water to avoid errors that can be caused by salts precipitated from the culture medium on the filter. Then, the filters were dried in a drying oven to constant weight. The increase in the microalgae biomass was determined by the difference in the masses of dry filters before and after filtration.

A stationary pH meter pH 213 (Hanna Instruments, Woonsocket Rhode Island, VA, USA) was used to determine the pH of microalgae suspensions. The densities of the suspensions were determined using a hydrometer. For this, a calibrated hydrometer was placed in a microalgae suspension and the density was determined on a scale with consideration for the suspension temperature. The viscosity of the suspensions was measured using a VPZh-2 capillary viscometer. The kinematic viscosity of a suspension of microalgae is equal to the product of the time of the suspension flowing through a capillary of a certain volume by the constant of the viscometer. The constant of the viscometer is independent of temperature and is determined only by the geometric dimensions of the viscometer.

### 4.4. Extraction of Lipids

Lipids were extracted using the Folch method [37]. For this, 2 mL of chloroform:methanol (2:1 by volume) mixture was used per 100 mg of dry biomass. Then, the sample was processed on a I100-34 ALT ultrasound scanner (Inlab, St. Petersburg, Russia) for 30 min to extract the lipid fraction. After sonication, 0.25 volume of 0.9% sodium chloride solution was added to the sample, and the mixture was vigorously stirred. LPC obtained from microalgae biomass was concentrated using a Heidolph vacuum rotary evaporator (DV-Ekspert, Moscow, Russia). LPC in a volume of 100 mL was placed in a 200 mL round-bottom flask. The concentration was carried out in a vacuum mode of 127 mbar, at 40 °C, for 30 min. The extract volume decreased by 30%.

After phase separation, the organic phase was removed and evaporated using a Heidolph vacuum rotary evaporator (DV-Expert, Moscow, Russia) to constant weight. The dry weight of the lipid fraction was determined by weighing. The lipid content **W_L_** (%) was determined by the following formula:

(1) $$W_{L}=\frac{m_{L}}{m_{B}}\cdot 100\%,$$

where $m_{L}$—the mass of extracted lipids; $m_{B}$—the mass of dry biomass.

### 4.5. Purification of the Microalgae Lipid Complex

The purification method of the lipid complex isolated from the microalgae biomass is based on the complete dissolution of the complex in *n*-hexane [4]. An organic solvent (*n*-hexane) was added to the obtained lipid complex after evaporation in a rotary evaporator, and the complex was dissolved therein at constant stirring. Then the sample was filtered and evaporated under vacuum in a Rotavapor R-300 rotary evaporator (Buchi Labortechnik AG, Flawil, Switzerland). The duration of the process of dissolution of the lipid–pigment complex and the intensity of stirring were varied within the course of the study. The transparency of the lipid complex was evaluated as follows. Solutions of formazin, hydrazine sulfate, and urotropine were prepared. An aqueous suspension of formazin diluted in a ratio of 1:1000, obtained by the interaction of equal volumes of an aqueous solution of hydrazine sulfate with a mass concentration of 10 g/dm^3^ and an aqueous solution of urotropine with a mass concentration of 100 g/dm^3^, was considered as a unit of the formazin scale. The mixture was left for 24 h at a temperature of (20 ± 2) °C to obtain a stable suspension. The resulting suspension was used to prepare formazin suspensions with turbidity of 50 FTU, 2 FTU. After measuring the optical density of the studied lipid complex, the degree of its transparency in formazin turbidity units was determined by the calibration graph. A PE-5400UF photocolorimeter (Ekros, Kazan, Russia), which allows measurements at wavelengths of 570 nm or 590 nm, was used. Based on the two obtained values of optical density, a linear graph was built: the degree of transparency–optical density. The microalgae lipid complex was placed (without air bubbles) in a photocolorimeter cuvette 20 mm long; the cuvette was quickly placed inside the instrument and the optical density was measured compared to the cuvette with the same lipid complex but filtered through a folded filter at a temperature of (20 ± 2) °C.

### 4.6. GC-MS Analysis

FAME content was determined to be analogous to [38,39] on chromatography system Agilent 7890 B with MS detector 5977. An identification of fatty acid methyl ester was curried out on NIST MS libraries. Transmethylation was carried out according to standard protocol in 1 g lipids in 5 mL dry hexane with 1 mL 5% MeONa in MeOH, at room temperature for 20 min. Prior to the reaction, toluene was added to the mixture to improve transmethylation of nonpolar lipids, and the tubes were filled with N_2_ gas to avoid oxidation. After the transmethylation, 5% NaCl aqueous solution and hexane were added in equal proportions, and the hexane phase was recovered after centrifugation. The hexane phase was used for GC-MS analysis. GC-MS parameters: sample was injected at a volume of 1 μL in split 1:50 mode by an Agilent 7693 autosampler into an Agilent 7890A gas chromatograph (Agilent, Santa Clara, CA, USA) equipped with a 30 m × 0.18 mm ID fused silica capillary column with a chemically bonded 0.15 μm HP-5 MS phase. The gas flow rate through the column was 1 mL min^−1^, and the column temperature was held at 80 °C for 3 min and increased to 280 °C in 20 °C min^−1^. The column effluent was introduced into the ion source of an Agilent 5977A. The transfer line and the ion source temperatures were 250 °C and 230 °C, respectively. Ions were generated by a 70 eV electron beam at an emission current of 35 μA. The acceleration voltage was turned on after a solvent delay of 120 s and the detector voltage was 1500–2000 V. All GC–MS data were analyzed with Chemstation software (Agilent, Santa Clara, CA, USA) with library NIST 2014.

### 4.7. Antibiotic Activity by Disc Diffusion Test

Antibiotic activity was determined by the disc diffusion test [40]. Discs with ampicillin (10 µm/disc) were used as control. Discs (6 mm) containing 20 μL of purified lipid extracts at different concentrations were placed on the surface of the agar culture medium. The forceps were sterilized over a burner flame, and the discs were removed one at a time. The discs were placed separately from each other, at a distance from the edge of the Petri dish. The bacteria were inoculated with a glass spatula into dishes with the medium. The dishes were incubated for 24 h at 37 °C, and the inhibition zone was measured in millimeters (mm); the disc diameter was subtracted from the inhibition zone [41].

To obtain a pure lipid–pigment complex from microalgae, organic solvents methanol and chloroform were used. *A. platensis* and *C. vulgaris* lipid complex samples, which included all isolated lipids, were tested at three concentrations of 5.0, 7.5, and 10.0 μ/disc. Discs with ampicillin (10 μ/disc concentration for each disc) were used as controls to assess the inhibition zones. The tests were carried out in triplicate (biological triplicates). Three different Gram-positive bacteria (*B. pumilus*, *L. mesenteroides*, *P. pentosaceus*) and one Gram-negative strain (*E. coli*) showed insignificant differences in the diameter of the inhibition zone when reacted with LPC isolated from microalgae. The antibacterial activity of microalgae was evaluated using various organic solvents that are involved in the efficient extraction of LPC (methanol and chloroform-methanol). According to the Folch method, organic solvents (methanol and chloroform-methanol) were used to extract lipid-containing fractions [42]. Lipids from microalgae and model solutions for plotting a calibration graph were extracted using Folch solution (chloroform:methanol = 2:1 by volume). Microalgae isolates (200 μL) were diluted with 800 μL of 0.9% NaCl, then the samples were extracted twice with two mL of Folch solution. The combined organic phase was left to settle for 24 h, after which it was centrifuged at 7000 rpm for more complete phase separation. The upper layer was carefully decanted and the lower layer was used for determination of lipids by IR spectroscopy. Aliquots of the extract with a volume of 50 μL were dried for 30 min on a zinc selenide substrate in a thermostat at 37 °C. IR absorption spectra were recorded on an FT-801 FT-IR spectrometer (Simeks, St. Petersburg, Russia) in the range 500–4000 cm^−1^. The spectra were recorded with 32 scans with a resolution of 4 cm^−1^.

The antibacterial activity of LPC isolated from the biomass of *C. vulgaris* and *A. platensis* was determined according to the following method. Test bacteria were Gram-positive strains *Bacillus pumilus* B-1133, *Leuconostoc mesenteroides* subsp. mesenteroides B-9280, *Pediococcus pentosaceus* B-7537, and Gram-negative bacteria *Escherichia coli*. These test strains are pathogenic and opportunistic bacteria, which are standard for determining the antibiotic activity of microorganisms. They grow well on nutrient media; form characteristic, often colored colonies; and are fairly easy to identify [43]. The cultivation was carried out in a Luria–Bertani nutrient medium, pH 8.5. LB agar medium included an additional 2.0 g/mL of agar. Storage temperature was 4 °C. Microalgae isolates in a volume of 100 μL were applied to a solid nutrient medium using a Drigalski spatula. Petri dishes with isolates were dried at 30 °C for 10 min. After 10 min, preprepared sterile paper discs were applied. Each disc was loaded with 10 μL of the studied microalgae isolates. Dry discs were used as negative controls, and antibiotic (ampicillin) discs were used as positive controls. The experiment was carried out in duplicate. The antibacterial activity of microalgae isolates was assessed by the presence and diameter of the lysis zone.

### 4.8. Minimum Inhibitory Concentration

The immunoassay 96-well plate was washed with ethanol before use. The first row of wells was filled with 50 μL of methanol as a positive control; the second row of wells was filled with 50 μL of LB culture medium and 50 μL of a bacterial suspension as a negative control. The third to seventh rows of the 96-well plate were filled with a lipid complex at various concentrations (10.0, 7.5, 5.0, 2.5 μg/disc) and inoculated with 50 μL of a bacterial suspension. The positive control was the solvent methanol, which was used to extract the lipid complex from *C. vulgaris* and *A. platensis*. Negative control was LB culture medium with Gram-positive (*B. pumilus*, *L. mesenteroides*) and Gram-negative (*E. coli*) strains. After 24 h, the sample was analyzed using a microplate reader (CLARIO star, BMG Labtech). The minimum inhibitory concentration value was defined as the lowest concentration of the lipid extract in the nutrient medium, which inhibits the growth of the test microorganism [44].

### 4.9. Lipid Complex Cytotoxicity

The cytotoxicity of microalgae lipid complexes was determined in relation to skin melanoma cell cultures SK-MEL-2 (ATCC^®^ HTB-68™). Cell lines were cultured in RPMI or DMEM with FBS in an incubator at 37 °C and 5% CO_2_ to ensure the growth and viability of cancer cells. Cytotoxicity was determined using MTT solution (3-(4,5-dimethylthiazolyl-2)-2,5-diphenyltetrazolium bromide) [37,45]. After incubation for 24 h, the cells were treated with various concentrations of *C. vulgaris* and *A. platensis* lipid complexes and incubated for 48 h. MTT solution (5 mg/mL) was added to each Petri dish and further incubated for 4 h at 37 °C. Dimethyl sulfoxide (DMSO)-supplemented medium was used as a control. Cells treated with doxorubicin (10 μg/mL) and untreated cells were used as positive control and negative control, respectively. IC50 was calculated for each cancer cell line using linear regression equations [46]. Each concentration of lipid complexes of microalgae was analyzed separately in triplicate.

The cancer cell lines were cultured for 24 h. Subsequently, the cells were exposed to lipid complexes of microalgae at concentrations corresponding to their IC50, and cell viability was assessed after 12, 24, 36, and 48 h. After 48 h treatment, the medium was replaced with fresh medium (without extract), and the cells were cultured for another 12 h and 24 h. Cell viability was determined by the conventional trypan blue exclusion method. For this, 20 μL of a suspension of the studied lipid complexes of microalgae *C. vulgaris* and *A. platensis* was mixed with 20 μL of a 0.2% trypan blue solution prepared in buffered saline (pH 7.4) with the addition of 0.02% (by volume) sodium azide, and the total number and the number of living cells were counted in Goryaev’s chamber [47]. Cells not treated with the lipid complex of microalgae were used as a control. The number of viable SK-MEL-2 cancer cells was assumed to be 100%. During the experiment, the following correlation was considered: 1 μg/mL = 10 μg/disc.

To analyze the cytotoxicity of lipid complexes of microalgae in vitro, 5 × 10^5^ cells were treated with *C. vulgaris* and *A. platensis* lipid complexes for 48 h in a 6-well plate, and then detached and placed in a new Petri dish with culture medium. After incubation for 6–24 h, the status and morphology of cell attachment was observed. As a control, cells were cultured in the same Petri dish without lipid complexes of microalgae. A double-staining assay with acridine orange/ethidium bromide was performed as described in [48]. Skin melanoma cell lines (SK-MEL-2) were treated with *C. vulgaris* and *A. platensis* lipid complexes, incubated for 48 h, trypsinized, and stained with acridine orange/ethidium bromide. Nikon TS100 microscope (KF Microscope Plus, St. Petersburg, Russia) was used to study cell suspensions at 400× magnification. The results were obtained in three independent determinations.

### 4.10. Antioxidant Activity of Microalgae Lipid Complexes

To study the antioxidant activity, the studied samples of lipid complexes obtained from the biomass of microscopic algae were dissolved in 1 mL of dimethyl sulfoxide and treated with ultrasound (Sonorex Super RK 100 H, Bandelin, Germany) for 5–10 min until their complete dissolution. Samples were dissolved immediately on the day of analysis. The antioxidant activity of the samples was determined by their ability to reduce the 2,2-diphenyl-1-picrylhydrazyl radical (DPPH, C_18_H_12_N_5_O_6_, MW = 394.33). The reaction of interaction of antioxidants with DPPH radical proceeds as follows:

DPPH* + AH → DPPH–H + A*.

As a result of the reduction of the DPPH radical with an antioxidant, the purple-blue color of DPPH in ethanol decreases, and the reaction is monitored by the change in optical density by the spectrophotometric method.

For the analysis, solutions of the studied samples of lipid complexes obtained from the biomass of microscopic algae were mixed with 2.85 mL of a freshly prepared 0.1 mM solution of 2,2-diphenyl-1-picrylhydrazil. The mixture was incubated in the dark at room temperature for 30 min. The decrease in optical density in comparison with the control (dimethyl sulfoxide solution) was recorded at 517 nm (UV-3600 spectrophotometer, Shimadzu, Japan). Ascorbic acid (AA) solutions of known concentration were used as standard solutions. The results of the analyses were expressed in mg AA equivalent per gram of extract or individual compound (mg AA/g). In the absence of sample weight, the antioxidant activity was expressed by the EC50 value in μL of the solution required to bind 50% of the DPPH radical. The antioxidant activity of the samples was analyzed in triplicate.

### 4.11. Statistical Analysis

The data were subjected to analysis of variance (ANOVA) using Statistica 10.0 (StatSoft Inc., 2007, Tesla, WV, USA). Post hoc analysis (Duncan’s test) was undertaken to identify samples that were significantly different from each other. The equality of the variances of the extracted samples was checked using the Levene test. Differences between means were considered significant when the confidence interval was smaller than 5% (*p* < 0.05).

## 5. Conclusions

The results showed that LPCs can play a crucial role as antibacterial agents since the purified lipid fraction inhibits the growth of Gram-positive (*B. pumilus*, *L. mesenteroides*, *P. pentosaceus*) and Gram-negative (*E. coli*) bacteria. It should be noted that LPC under experimental conditions was most effective against *P. pentosaceus* among Gram-positive bacteria. The MIC value for Gram-positive bacteria was 3.0 μg/disc, while for Gram-negative bacteria it was 2.0 μg/disc. Antimicrobial activity is directly related to the LPC concentration. The cytotoxic and antioxidant activities of samples of lipid complexes of microalgae *C. vulgaris* and *A. platensis* were proven, and their physicochemical properties and fatty acid composition were studied. When studying the effect of the lipid complex of microalgae on the growth activity of SK-MEL-2 skin melanoma cancer cells stained with acridine orange/ethidium bromide, a significant inhibition of cancer cell growth was observed. The ability of tumor cells to form colonies decreased by at least 50%. According to these results, *C. vulgaris* and *A. platensis* microalgae lipid complex samples have the potential to inhibit dual tumor colonization in vitro compared to normal cells. The lipid–pigment complex of microalgae demonstrated the presence of cytotoxic activity and inhibition of cancer cell proliferation. Based on the results, it can be concluded that LPCs isolated from microalgae have antimicrobial potential; the purified LPC can become the basis for an alternative preparation for the prevention of microbial contamination of feed. Based on the findings of studies of the physicochemical and biological properties of microalgae, an investigation is planned on the feasibility of using Baltic Sea microalgae as a promising raw material for a variety of practical applications (bioremediation, raw material for fuel production, bioactive compounds, fertilizers, and animal feed), which could be in demand in the food, pharmaceutical, and cosmetic industries.

## Figures and Tables

**Table 1:** Physicochemical properties of microalgae samples.

Indicator	Microalgae	
	*C. vulgaris*	*A. platensis*
Duration of cultivation, days	7	7
Optical density (OD_750_)	0.59 ± 0.01	0.86 ± 0.02
pH	8.7 ± 0.25	11.6 ± 0.34
Density, kg/m^3^	909.34 ± 27.42	928.48 ± 27.75
Dynamic viscosity, 10^−3^ Pa·s	1.13 ± 0.03	0.96 ± 0.02
Dry matter content,%	0.49 ± 0.01	0.59 ± 0.01
Color	green	green
Odor	faint herbaceous	faint herbaceous

**Table 2:** Qualitative and quantitative content of the lipid fraction of the lipid complex of microalgae samples.

Lipids, %	Microalgae	
	*C. vulgaris*	*A. platensis*
Neutral lipids	37.1 ± 0.5	59.3 ± 0.9
Triacylglycerides	13.4 ± 0.4	28.7 ± 0.4
Fatty acids	24.3 ± 0.2 *	27.9 ± 0.4 *
Polar lipids ^1^	0.9 ± 0.1 *	1.1 ± 0.2 *
Unsaponifiable substances	14.1 ± 0.2	18.4 ± 0.4
Chlorophyllides ^2^	16.3 ± 0.1	6.3 ± 0.2
Other impurities ^3^	57.9 ± 0.8	16.2 ± 0.3

Values in a row followed by the symbol “*” do not differ significantly (*p* > 0.05) as assessed by the post hoc test (Duncan’s test). Average values are presented (n = 3). ^1^ Polar lipids in this table are glycolipids and phospholipids quantified by HPLC with the mass fraction of chlorophyll phytol side chains included in the unsaponifiables. ^2^ Chlorophyllide (non-phytol fragment of chlorophylls) is calculated based on the assumption that all chlorophyll pigments had the same molecular structure as chlorophyll a. ^3^ “Other impurities” is the difference between 100% and known components.

**Table 3:** Fatty acid composition of the lipid fraction of the lipid complex of microalgae samples.

Content, mg/g	Microalgae	
	*C. vulgaris*	*A. platensis*
Myristic acid	0.21 ± 0.1	0.14 ± 0.1
Palmitic acid	4.13 ± 0.2 *	6.39 ± 0.2 *
Oleic acid	6.64 ± 0.2	5.33 ± 0.2
Stearic acid	11.68 ± 0.2	11.16 ± 0.2
Linoleic acid	3.72 ± 0.2	3.95 ± 0.2

Values in a row followed by the symbol “*” do differ significantly (*p* > 0.05) as assessed by the post hoc test (Duncan’s test). Average values are presented (n = 3).

**Table 4:** Antimicrobial activity (zone of inhibition, mm) of the *C. vulgaris* lipid complex.

Sample	Concentration of the Lipid Complex, μg/disc		
	5.0	7.5	10.0
Control	20.0 ± 0.15 a	20.0 ± 0.15 a	20.0 ± 0.15 a
*E. coli*	6.1 ± 0.13 b	16.3 ± 0.23 b	17.0 ± 0.47 b
*B. pumilus*	6.2 ± 0.23 b	14.9 ± 0.13 b	16.0 ± 0.27 b
*L. mesenteroides*	6.6 ± 0.14 b	14.5 ± 0.15 b	17.0 ± 0.65 b
*P. pentosaceus*	6.6 ± 0.24 b	15.5 ± 0.23 b	18.0 ± 0.45 ab

Control—ampicillin (10.0 μg/disc). Values in a row followed by the same letter do not differ significantly (*p* > 0.05) as assessed by the post hoc test (Duncan’s test). Data presented as a mean ± SD (*n* = 3).

**Table 5:** Antimicrobial activity (zone of inhibition, mm) of the *A. platensis* lipid complex.

Sample	Concentration of the Lipid Complex, μg/disc		
	5.0	7.5	10.0
Control	20.0 ± 0.15 a	20.0 ± 0.15 a	20.0 ± 0.15 a
*E. coli*	7.0 ± 0.23 b	14.4 ± 0.45 b	17.0 ± 0.21 b
*B. pumilus*	8.0 ± 0.14 b	14.0 ± 0.15 b	16.0 ± 0.22 b
*L. mesenteroides*	7.6 ± 0.14 b	15.6 ± 0.23 b	18.0 ± 0.24 ab
*P. pentosaceus*	6.3 ± 0.36 b	15.2 ± 0.12 b	18.0 ± 0.45 ab

Control—ampicillin (10.0 μg/disc). Values in columns followed by the same letter do not differ significantly (*p* > 0.05) as assessed by the post hoc test (Duncan’s test). Data presented as a mean ± SD (*n* = 3).

**Table 6:** Antioxidant activity of lipid complex samples obtained from the microscopic algae biomass.

Lipid Complex	Antioxidant Activity, mg AA/g
*C. vulgaris*	45.92 ± 0.77
*A. platensis*	45.85 ± 0.77

Data presented as a mean ± SD (*n* = 3).

## Data Availability

The raw data supporting the conclusions of this article will be made available to any qualified researcher on request.
